# Loss of Reduction and Malunion After Cortical Perforation During Flexible Nailing of an Open Tibia Fracture

**DOI:** 10.7759/cureus.28750

**Published:** 2022-09-03

**Authors:** Justin Aflatooni, Andrew George, Aharon Z Gladstein

**Affiliations:** 1 Orthopedics and Sports Medicine, Houston Methodist Hospital, Houston, USA; 2 Orthopedic Surgery, Texas Children's Hospital, Houston, USA

**Keywords:** loss of reduction, open tibia fracture, flexible intramedullary nail, malunion, tibia shaft fracture

## Abstract

Tibial shaft fractures have a relatively high incidence in the pediatric population. There are numerous ways to address this trauma including external fixation, plate osteosynthesis, flexible nailing, and closed treatment with the selection of each modality depending on multiple factors, including fracture characteristics as well as potential adverse events. Flexible nailing is a method of treatment at our institution for displaced tibial shaft fractures in patients who are not obese, who are skeletally immature, and whose fractures are not amenable to closed treatment. One of the described complications of this treatment method is an angular deformity. In this case report, we present a valgus recurvatum malunion of a pediatric left open tibia and fibula diaphyseal shaft fracture in a 13-year-old female due to an accidental bicortical perforation of one of the nails without concomitant fixation of the fibula. The purpose of this paper is to present a surgical complication and how to avoid it.

## Introduction

Tibial shaft fractures have a relatively high incidence in the pediatric population with some reports as high as 20% of inpatient pediatric orthopedic trauma [1]. There are numerous ways to address this trauma including external fixation, plate osteosynthesis, flexible nailing, and closed treatment. The selection of each modality depends on several factors, including fracture characteristics and potential adverse events. Flexible nailing is a common treatment method for relatively stable fracture patterns in patients who are skeletally immature as it offers relative stability with the maintenance of alignment while protecting the physes and minimizing soft tissue morbidity [1,2]. While external fixation has been associated with higher complication rates than other treatments, complications from flexible nailing of tibial shaft fractures, even in the context of an open injury, are rare [1-4]. In this case report, we present a valgus recurvatum malunion of a pediatric left open tibia and fibula diaphyseal shaft fracture in a 13-year-old female, due to an accidental bicortical perforation of one of the nails.

## Case presentation

**Figure 1:**
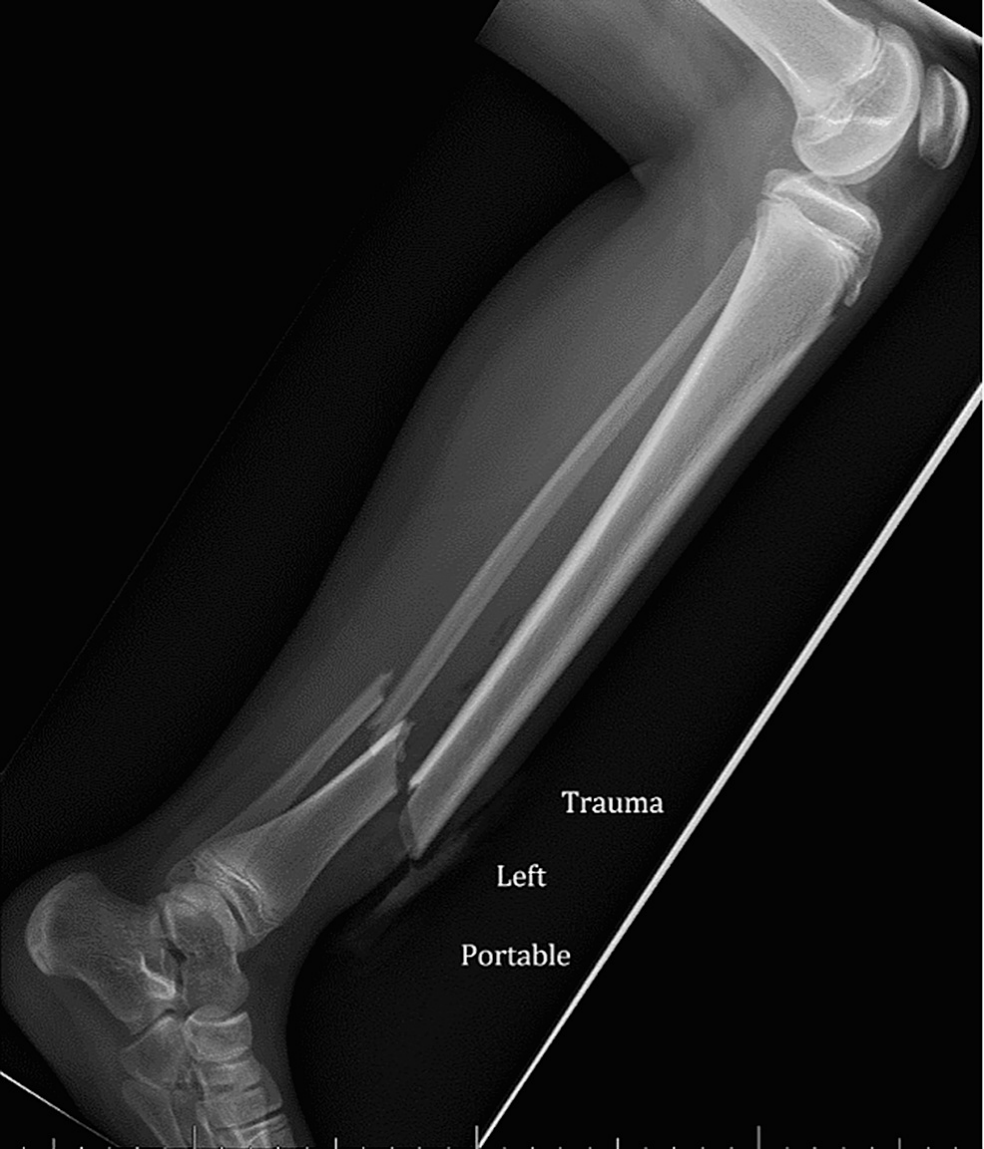
Lateral tibia-fibula radiographs of initial open tibia/fibula shaft fractures.

**Figure 2:**
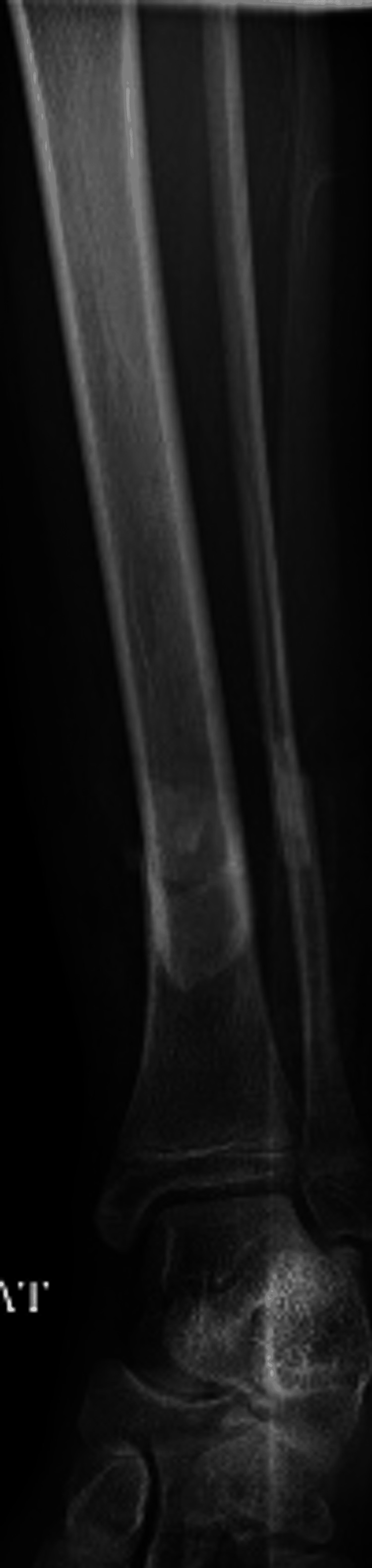
Anterior-posterior tibia-fibula radiographs of initial open tibia/fibula shaft fractures.

A 13-year-old female with no significant medical history presented to our emergency department with an open Gustilo-Anderson type 2 left tibial shaft fracture after falling off an all-terrain vehicle (ATV). On exam, the patient had a 1 cm wound overlying the anterior distal tibia with bony protrusion. There was no gross contamination, neurovascular deficit, or concern for evolving compartment syndrome. Her height and weight were 150 cm and 41 kg, respectively (BMI 18). Radiographs demonstrated a displaced distal tibia and fibula fracture that was 2 cm shortened (Figures 1, 2).

 

Antibiotics were administered in the emergency department, and she was placed in a long leg splint. She was admitted overnight and scheduled for irrigation and debridement, as well as fracture stabilization with flexible intramedullary nails the following day. 

**Figure 3:**
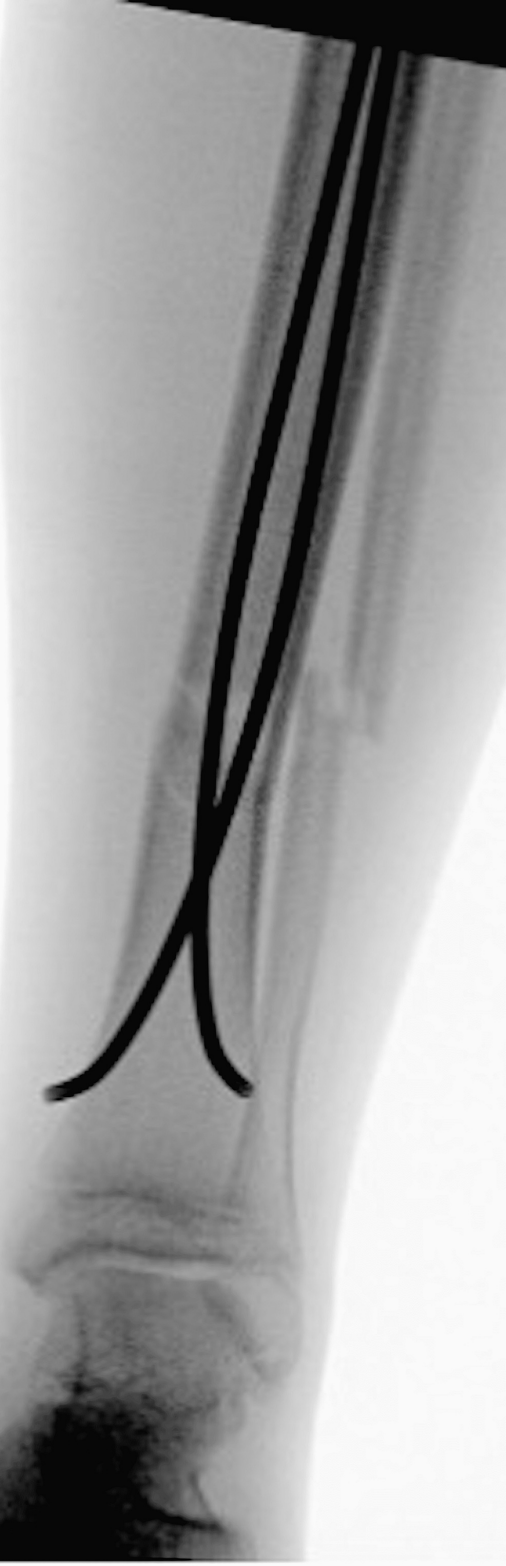
Anterior-posterior tibia-fibular intra-operative radiographs of irrigation and debridement with concomitant flexible nailing of the tibial shaft.

**Figure 4:**
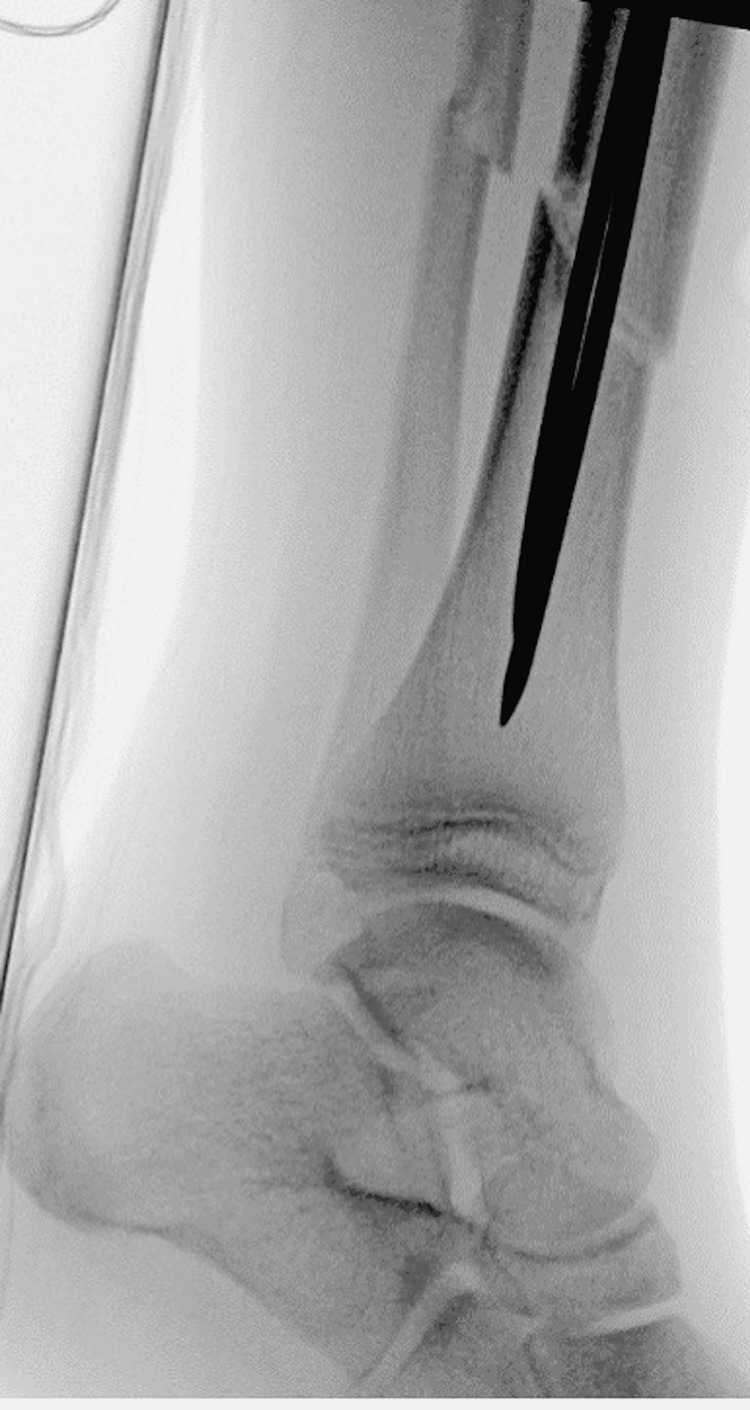
Lateral tibia-fibula intra-operative radiographs of irrigation and debridement with concomitant flexible nailing of the tibial shaft.

In the operating room, the open wound was irrigated with three liters of normal saline. Under fluoroscopy, we localized our starting point for the first flexible nail after marking the proximal tibial physis. We then used an awl to create our entry site approximately 2 cm distal to the physis. We passed the first flexible nail down the tibial shaft just proximal to the fracture site. Similarly, we used the awl to create the entry site for the second flexible nail and, again, drove this down to just proximal to the fracture site. We then reduced the fracture with traction and translation and subsequently passed the two nails across the fracture site after appropriate alignment was obtained. The nails were cut proximally and tamped under the skin nearly flush with the bony cortex. Final fluoroscopic shots revealed the medial nail had perforated the distal tibial cortex (Figures 3, 4).

 

The fracture was stable to stress intra-operatively, and the decision was made to not revise the medial nail or to fix the fibular fracture, due to the stability of the tibia under stress. The proximal entry site wounds were irrigated and closed and a bivalved short leg cast was applied. 

**Figure 5:**
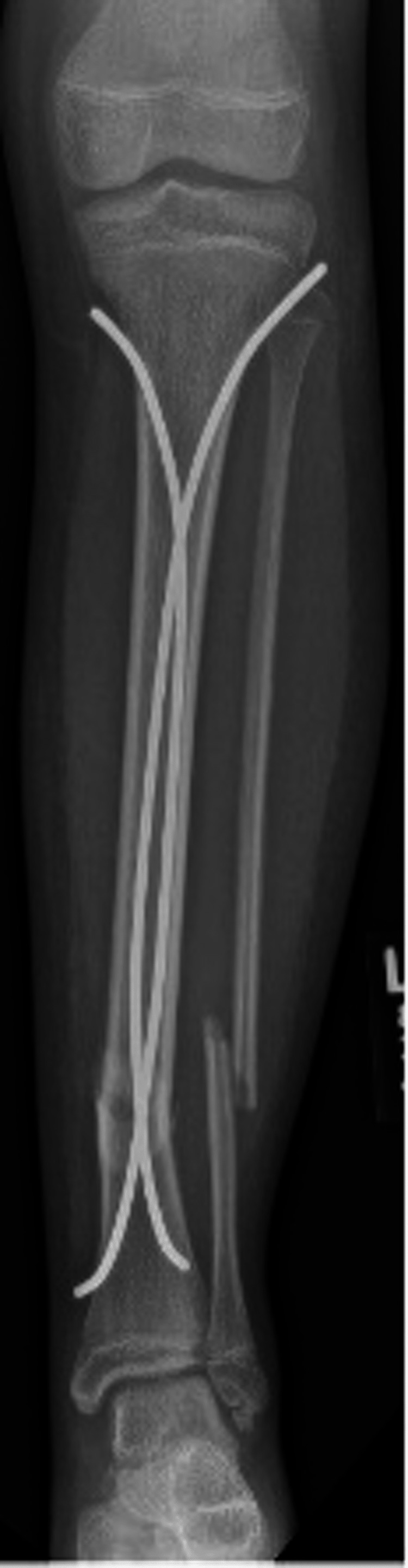
One-month post-operative anterior-posterior tibia-fibula radiographs of flexible nailing of the tibia.

**Figure 6:**
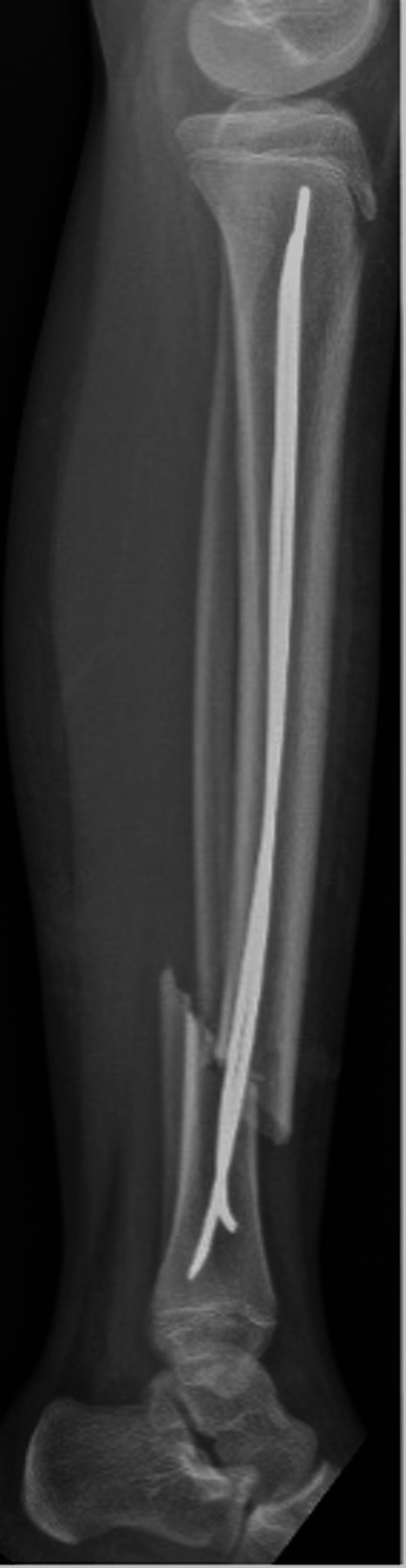
One-month post-operative lateral tibia-fibula radiographs of flexible nailing of the tibia.

Post-operatively, the patient remained non-weightbearing for four weeks. Radiographs at four weeks follow-up demonstrated a valgus malalignment, as well as shortening of the tibia and fibula fractures (Figures 5, 6).

 

**Figure 7:**
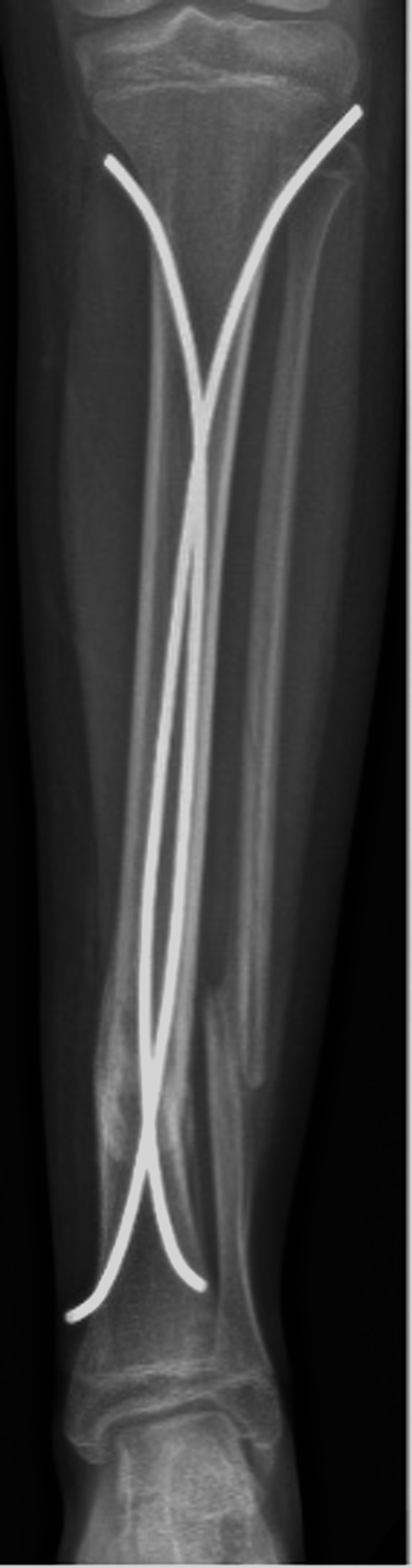
Three-month post-operative anterior-posterior tibia-fibula radiographs of flexible nailing of tibial shaft.

**Figure 8:**
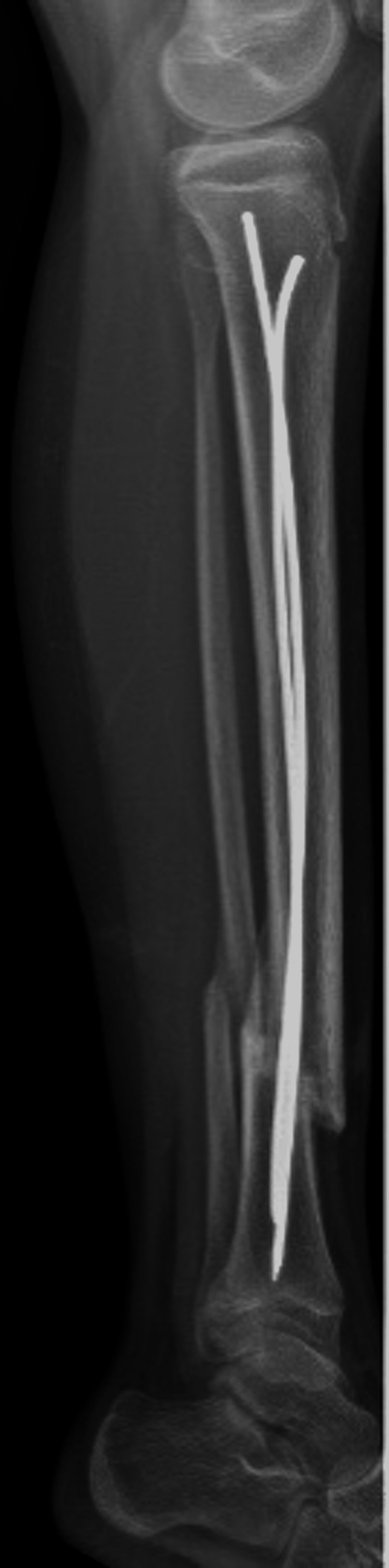
Three-month post-operative lateral tibia-fibula radiographs of flexible nailing of tibial shaft.

She denied pain and her incisions had healed well. Revision surgery was deferred as the fracture was still in acceptable alignment with mild valgus angulation (10°). Toe-touch weightbearing was initiated at that time. At the two-month mark, she continued to do well without pain or other concerns and radiographs demonstrated stable 10° valgus angulation with continued fracture site healing and so she was advanced to weight bearing as tolerated. Three-month post-operative radiographs demonstrated further healing of the fractures with persistent and worsening valgus malalignment (16°) (Figures 7, 8). She continued to deny pain, but the resulting deformity was bothersome to her and her family and she continued to rely on crutches for ambulation. Revision surgery with open reduction and internal fixation (ORIF) was indicated due to this deformity, which was appreciable both radiographically and clinically. 

 

During the revision surgery, the previous tibial incision scars were each opened and excised. The tibial nails were retrieved through the incisions and removed in their entirety. An anterior incision was made lateral to the tibial crest about the fracture site, and the periosteum was elevated over the fracture site to expose the bone. The healed fracture plane was still evident. A curved osteotome and rongeur were used to perform an osteotomy at the previous fracture site. Fibular fracture callus inhibited proper tibial reduction and so an additional incision over the previous fibular fracture site was made. Dissection was carried down to the fibula after identification and protection of the superficial peroneal nerve. Again, a rongeur and osteotome were used to osteotomized the fibula.

After the fibular osteotomy, the tibia and fibula were then able to be satisfactorily reduced, and the tibia was provisionally pinned with a K wire and fixed with a lag screw from anterior to posterior. A six-hole pre-contoured plate was then applied to the tibia and achieved excellent stability with no fibula movement under stress. We decided to forego fibular osteotomy fixation given our construct strength.

**Figure 9:**
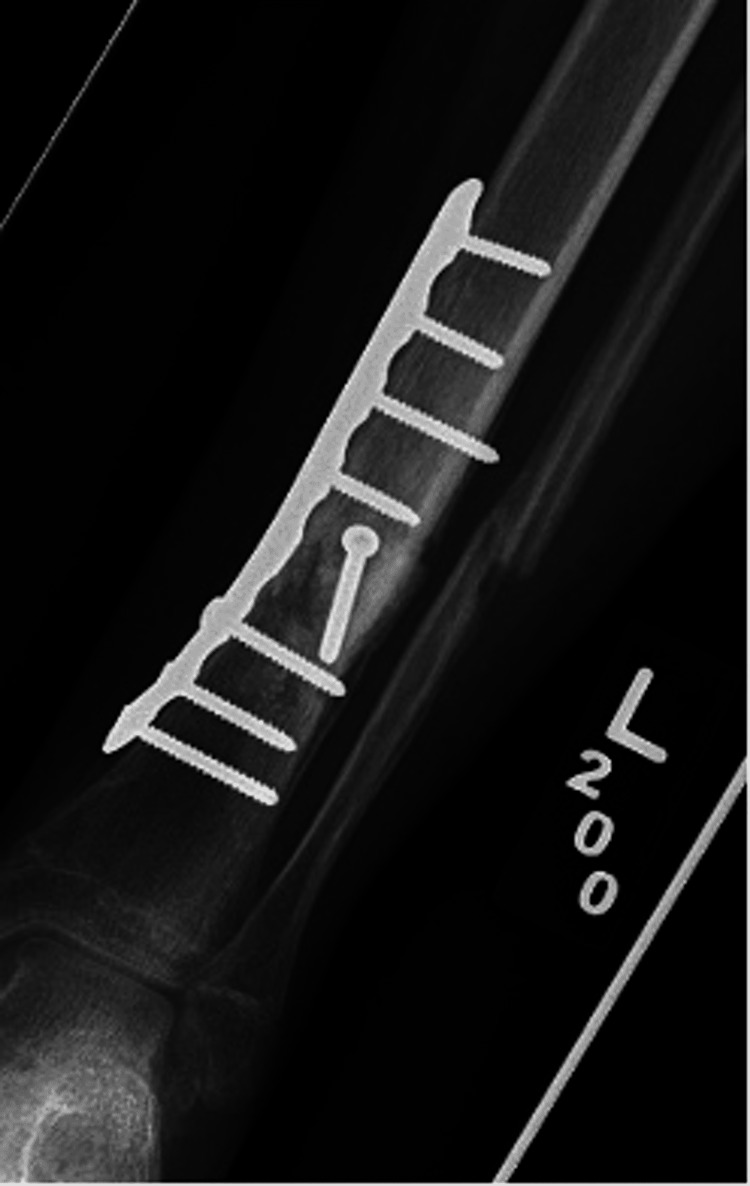
Post-operative anterior-posterior tibia-fibula radiographs of revision open reduction and internal fixation with removal of flexible nails of tibial shaft.

**Figure 10:**
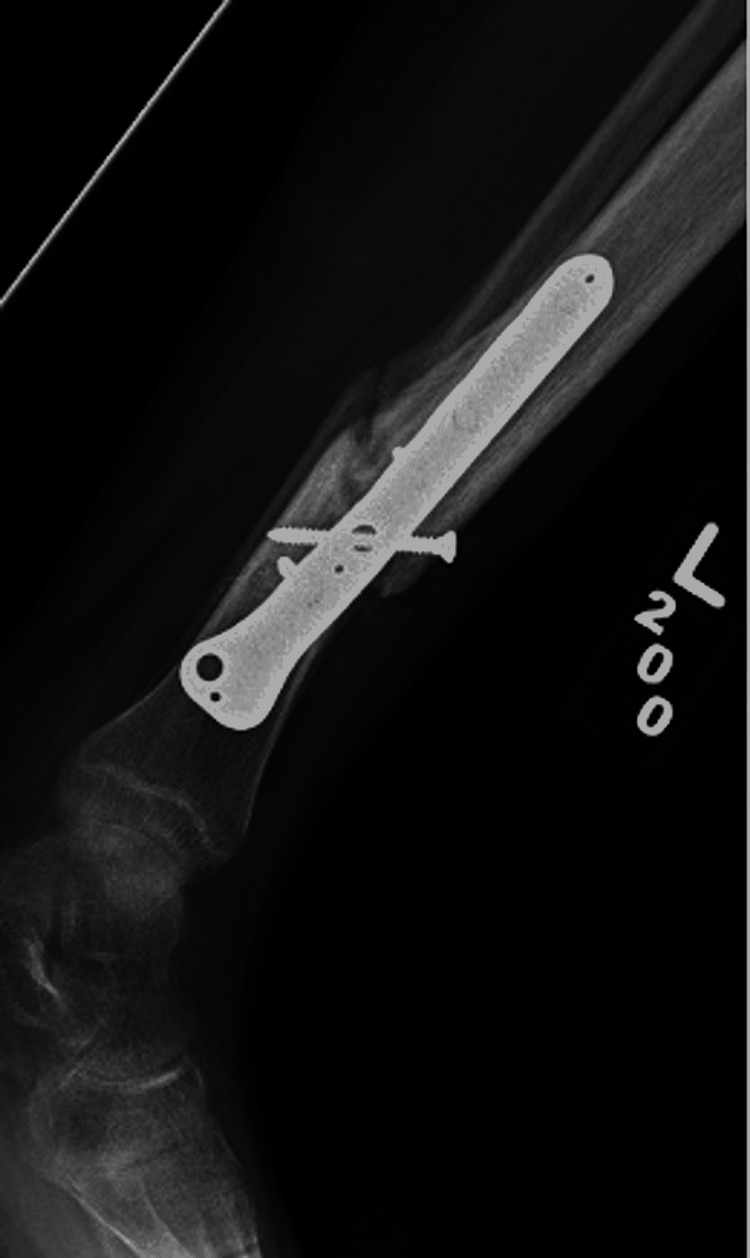
Post-operative lateral tibia-fibula radiographs of revision open reduction and internal fixation with removal of flexible nails of tibial shaft.

Fluoroscopy in multiple planes showed the excellent position of the hardware with a good reduction of the fracture. The wounds were irrigated and closed, and a bivalved short leg cast was re-applied. Post-operatively, the patient did well without residual pain or deformity. At the one-month mark, incisions had healed, and she denied pain. Radiographs demonstrated improved alignment and healing at both osteotomy sites (Figures 9, 10). She returned to full weight bearing.

 

## Discussion

In general, flexible nailing of the tibia is well tolerated and complications are rare. Some studies suggest that more favorable outcomes may be attained with cast augmentation [1,5]. Regarding the nails, one study discusses their placement to minimize adverse events. They emphasize achieving 80% canal fill with two equally sized, pre-bent nails inserted at the medial and lateral aspects of the proximal tibial metaphysis [2]. The cause of the angular deformity was likely distal cortical perforation of the medially inserted nail, combined with the obliquity of the fracture site. The resultant bicortical perforation of the medial nail effectively negated its effect and left the lateral nail to exert its valgus force on the tibia, unopposed. Furthermore, without the fibula operatively addressed, there was no buttress to resist this coronal deformation. Bonnevaille et al. noted that biomechanically, fixation of the fibula does augment the stability of tibial constructs, but also noted that there is no consensus on the need for fibular repair in every instance from a risk vs. benefit standpoint [6]. Nonetheless, fibular fixation at the index surgery may have altered the outcome in this case.

Mashru et al. discuss radiographic deformity parameters to help guide surgical management. They note that up to 10° of valgus, 5° of varus, 1 cm of shortening, and 10° of sagittal angulation may be tolerated. Further deformity affects reliable bony remodeling [7]. Our patient’s primary complaints were the appearance of the leg and the palpable bony deformity. Silva et al. observed that early weight bearing in low-energy tibial shaft fractures treated with closed reduction may decrease time to union [8]. While serial radiographs did show some callus formation, they did not impart a picture of hypertrophic callus formation of the tibia that one might expect with increased movement at the fracture site. Some studies note that fracture healing complications were seen more in Gustilo-Anderson type two and three fractures than with open type one fractures [2,9]. There may have been significant periosteal stripping resulting in slower healing than expected with a closed injury. However, the perforation of the distal tibial cortex was likely the most significant factor leading to fixation failure in this patient.

## Conclusions

The most important factor leading to treatment failure for this patient was a failure to recognize the consequence of perforation of the distal tibial cortex by the medial nail. Careful attention to this technical detail can help surgeons avoid the loss of fixation that led to the malunion of this fracture. Therefore, a thorough evaluation of final intra-operative radiographs should be performed with fixation issues addressed at that time.
